# Synthesis, Inhibitory Effects on Nitric Oxide and Structure-Activity Relationships of a Glycosphingolipid from the Marine Sponge *Aplysinella rhax* and Its Analogues 

**DOI:** 10.3390/molecules16010637

**Published:** 2011-01-17

**Authors:** Yuzo Fujita, Naohiro Ohshima, Ai Hasegawa, Frank Schweizer, Tadahiro Takeda, Fumiyuki Kiuchi, Noriyasu Hada

**Affiliations:** 1 Faculty of Pharmacy, Keio University, 1-5-30 Shibakoen, Minato-ku, Tokyo 105-8512, Japan; 2 Department of Chemistry and Medical Microbiology, University of Manitoba, Winnipeg, Manitoba, R3T 2N2, Canada

**Keywords:** glycosphingolipid, *Aplysinella rhax*, D-fucose, nitric oxide

## Abstract

The novel glycosphingolipid, β-D-GalNAc*p*(1→4)[α-D-Fuc*p*(1→3)]-β-D-GlcNAc*p*(1→)Cer (**A**), isolated from the marine sponge *Aplysinella rhax* has a unique structure, with D-fucose and *N*-acetyl-D-galactosamine moieties attached to a reducing-end *N*-acetyl-D-glucosamine through an α1→3 and β1→4 linkage, respectively. We synthesized glycolipid **1** and some non-natural di- and trisaccharide analogues **2**-**6 **containing a D-fucose residue. Among these compounds, the natural type showed the most potent nitric oxide (NO) production inhibitory activity against LPS-induced J774.1 cells. Our results indicate that both the presence of a D-Fucα1-3GlcNAc-linkage and the ceramide aglycon portion are crucial for optimal NO inhibition.

## 1. Introduction

**Figure 1:**
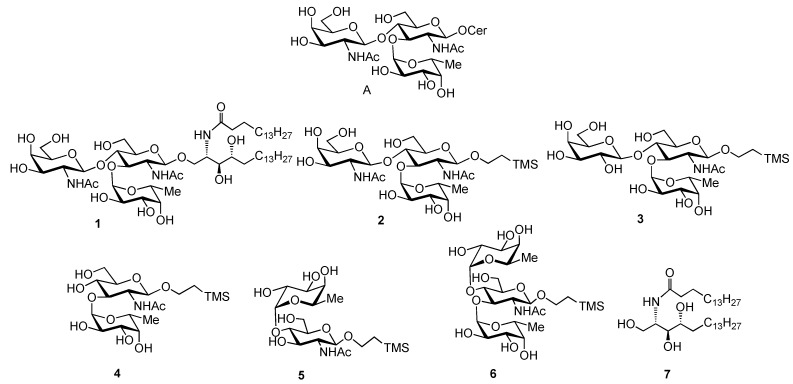
Target glycosphingolipid and the analogue compounds

Carbohydrates in the form of glycoconjugates, for example glycoproteins, glycolipids and proteoglycans, play an important role in many intracellular and extracellular events including cell-cell adhesion, cell differentiation, signal transduction, cancer metastasis and immune responses [1]. The majority of these studies have focused on higher animals and relatively little is known about the functions of glycoconjugates in lower animals [2]. In order to study the biological properties of glycans in glycoconjugates, over the past decade we have synthesized novel glycolipid and glycoprotein derivatives found in various invertebrates [3,4,5,6,7,8,9,10,11,12]. Organic synthesis is a powerful method to explore structure activity relationships by providing access to large amounts of homogeneous and structurally defined oligosaccharides including not only natural compounds, but also non-natural compounds [13]. Recently, Zollo et al. isolated and characterized a novel neutral glycosphingolipid (**A**, Figure 1) from the marine sponge *Aplysinella rhax* which features a D-fucose and an *N*-acetyl-D-galactosamine attached to a reducing-end *N*-acetyl-D-glucosamine through a α1→3 and a β1→4 linkages, respectively [14]. This was the first report on glycolipids containing D-fucose. Furthermore, these glycolipids have been found to exhibit significant inhibitory activity on LPS-induced nitric oxide (NO) release by J774.1 macrophages. In order to study the structure-activity relationships of these compounds inhibiting NO release, we previously reported the synthesis of β-D-GalNAc*p*(1→4)[α-D-Fuc*p*(1→3)]-β-D-GlcNAc*p*(1→)aglycon trisaccharide analogues, containing a 2-branched fatty alkyl residue and a 2-(trimethylsilyl)ethyl (TMS-Et) residue, respectively [6]. Moreover, biological evaluation of these novel glycosphingolipid analogues using an LPS-induced NO release assay demonstrated that the presence of D-fucose is crucial for the NO inhibitory effect, while structural modifications at the aglycon moiety appeared to have little to no effect on LPS-induced NO release [6]. In this study, we describe for the first time the total synthesis of glycosphingolipid **1** and its structural analogues **3**-**6** to elucidate the structure activity relationships on LPS-induced NO production in more detail (Figure 1). 

2-(Trimethylsilyl)ethyl β-D-galactopyranosyl-(1→4)-[α-D-fucopyranosyl-(1→3)]-2-acetamido-2-deoxy-β-D-glucopyranoside (**3**) was selected to explore how the presence of a terminal β-D-galacto-pyranosyl linkage instead of a 2-acetamido-2-deoxy-β-D-galactopyranosyl linkage affects the biological effect. Disaccharide-based regioisomers **4** and **5 **were selected to explore differences in the connectivity of the α-D-fucopyranosyl moiety to the-β-D-GlcNAc portion while trisaccharide **6** was chosen to study the effect of two α-D-fucopyranosyl linkages linked to the core -D-GlcNAc moiety. The NO-inhibitory affect of commercially available ceramide **7** and known trisaccharide **2 **was included in these experiments for comparison.

## 2. Results and Discussion

### 2.1. Chemical synthesis

**Scheme 1:**
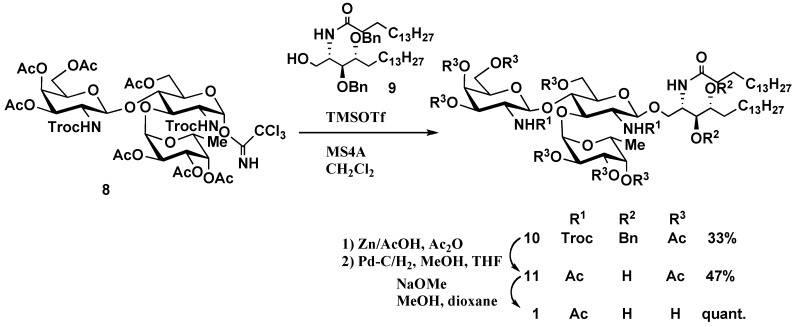
Synthesis of glycosphingolipid **1.**

*Synthesis of glycosphingolipid* **1**: Glycosylation of phytoceramide acceptor **9** [15] with the glycosyl imidate **8** [6] was carried out in the presence of trimethylsilyl trifluoromethanesulfonate (TMSOTf) [16] and 4 Å molecular sieves (MS4 Å) to obtain the desired glycolipid derivative **10 **in 33% yield with complete β-steroselectivity. Deprotection of the Troc group was achieved with Zn in a mixture containing acetic anhydride and acetic acid, followed by catalytic hydrogenolysis over 10% Pd/C in MeOH/THF to provide **11** in 47% yield. Deacetylation of **11 **using Zemplén conditions and purification by column chromatography on Sephadex LH-20 afforded target glycolipid **1 **quantitatively (Scheme 1). 

**Scheme 2:**
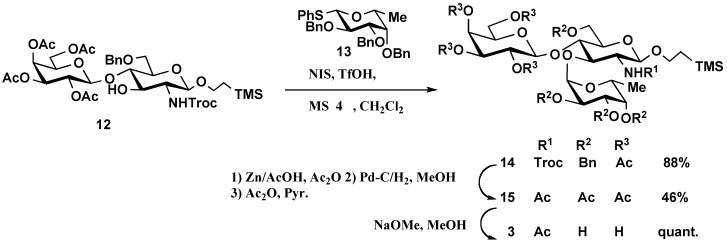
Synthesis of oligosaccharide **3.**

*Syntheses of oligosaccharides* **3**-**6: **Glycosylation of known disaccharide acceptor **12** [3] with the known D-fucopyranosyl donor **13** [6] in the presence of *N*-iodosuccinimide (NIS), trifluoro-methanesulfonic acid (TfOH) [17] and MS4 Å in dichloromethane provided the desired α-glycoside **14 **in 88% yield with complete α-stereoselectivity. The newly formed α-glycosidic linkage was confirmed by ^1^H-NMR spectroscopy. The anomeric proton of the fucose moiety in **14** appeared at 4.85 ppm as a doublet with a homonuclear proton-proton coupling constant of 3.7 Hz (H-1 of Fuc, δ = 4.85 ppm, *J*_H1,H2_ = 3.7 Hz). Deprotection of the Troc group in **14** was achieved with Zn in a mixture containing acetic anhydride and acetic acid, followed by catalytic hydrogenolysis over 10% Pd-C in MeOH and acetylation to provide **15**. Zemplén deacetylation and purification by column chromatography on Sephadex LH-20 produced trisaccharide **3 **quantitatively (Scheme 2). 

**Scheme 3:**
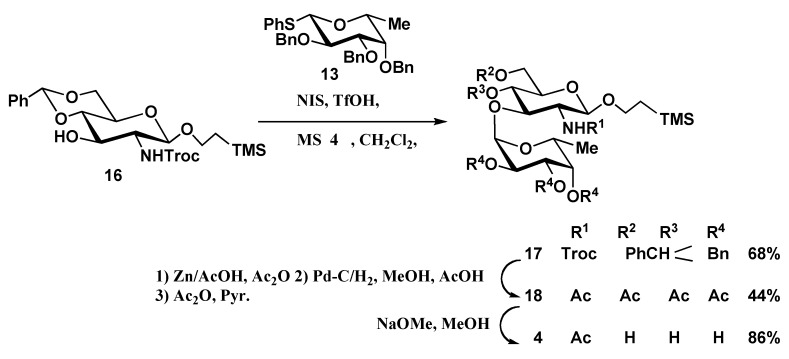
Synthesis of oligosaccharide **4.**

**Scheme 4:**
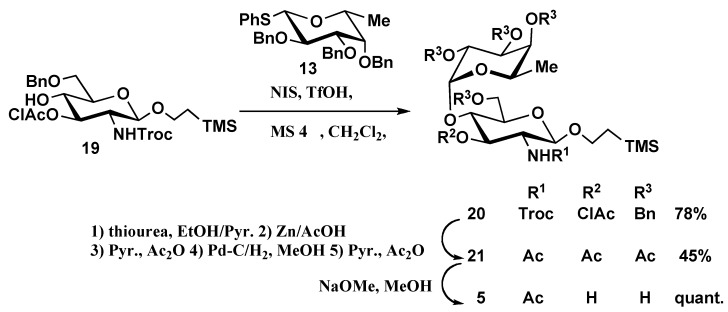
Synthesis of oligosaccharide **5**

**Scheme 5:**
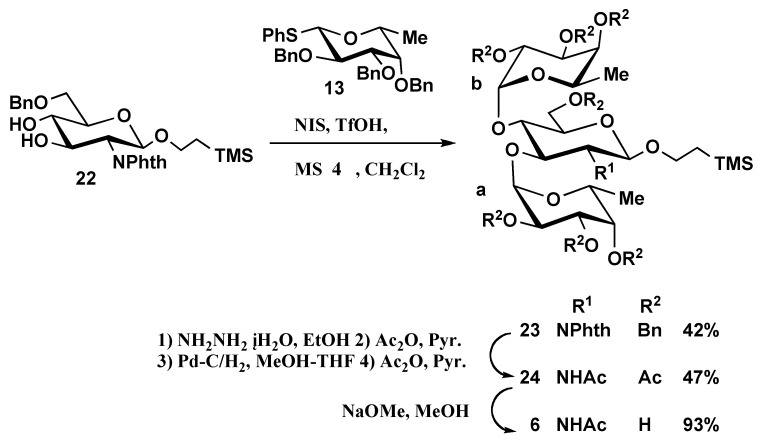
Synthesis of oligosaccharide **6**

The synthesis of disaccharides **4 **and **5 ** and trisaccharide **6** is outlined in Scheme 3, Scheme 4, Scheme 5. Glycosylation of known glycosyl acceptors **16, 19 [6] **and **22 [18]** with the D-fucopyranosyl donor **13** in the presence of NIS, TfOH and MS4 Å in dichloromethane gave the desired α-glycosides **17 **(68%), **20** (78%) and the trisaccharide **23** (42%) with complete α-steroselectivity, respectively. The newly formed α-glycosidic linkage was confirmed by ^1^H-NMR spectroscopy. The Troc-protecting group of **17** was converted into an acetamido group by reduction with Zn-AcOH followed by debenzylidenation and debenzylation with catalytic hydrogenolysis over 10% Pd/C in MeOH-AcOH and acetylation to afford **18 **in 44% yield. Finally, standard deacetylation and purification by column chromatography on Sephadex LH-20 furnished disaccharide **4 **in 86% yield (Scheme 3). 

Disaccharide **5** was synthesized from the disaccharide **20** in a six steps deblocking/blocking procedure (Scheme 4). At first, the chloroacetyl protecting group in **20** was deblocked with thiourea in an ethanol/pyridine solvent mixture before conversion of the Troc group into an acetamido group using standard conditions. Debenzylation using catalytic hydrogenolysis followed by acetylation provided protected disaccharide **21 **in 45% yield which was deprotected using standard conditions to provide disaccharide **5** (Scheme 4). 

The remaining trisaccharide **6** was prepared from protected trisaccharide **23 **in a five steps procedure. Initially, the phthalimido-protecting group of **23** was removed using hydrazine monohydrate in ethanol followed by standard acetylation, debenzylation by catalytic hydrogenation over 10% Pd-C in MeOH-THF and acetylation to provide peracetylated trisaccharide **24 **in 47% yield. Finally, standard deacetylation and purification by column chromatography on Sephadex LH-20 provided disaccharide **6** (Scheme 5). Oligosaccharide **2** was prepared according to a procedure previously reported by us [6].

### 3.2.Inhibitory Effects of Synthetic Compounds on NO Production

**Figure 2:**
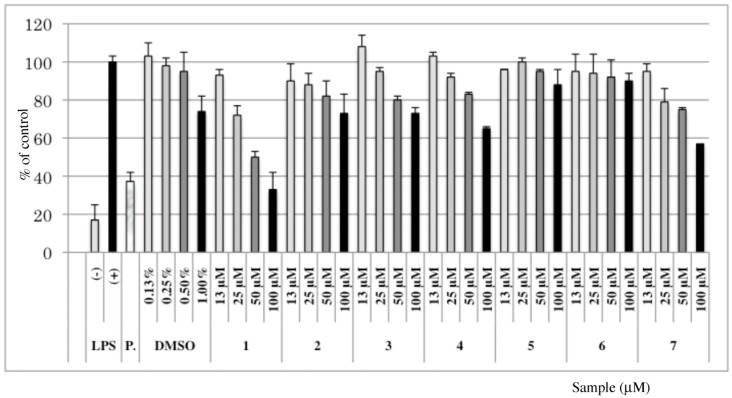
Inhibitory effects on NO production in LPS-activated J774.1 cells of compounds **1**-**7**. Each data represents the mean ± SD for quadruplet experimentals.P: Positive control (L-NMMA), 50 μM.

The synthetic compounds were evaluated for their ability to inhibit nitric oxide (NO) production by LPS-induced macrophage-like J774.1 cells [19] (Figure 2). NO, a short living mediator is synthesized by a family of enzymes termed NO-synthase. Two types of NOS are recognized: constitutive isoforms and inducible isoforms (iNOS). iNOS is regulated by inflammatory mediators (LPS, cytokines) and the excessive production of NO by iNOS has been implicated in the pathogenesis of the inflammatory response [14]. The glycolipid **1** showed comparable NO inhibitory activity in high concentration (100 μM) to N^G^-monomethyl-L-arginine (L-NMMA) used as the positive control. Related compounds having the common D-Fucα1-3GlcNAc structure (*i.e*. **2**, **3** and **4**) also showed significant inhibitory activity resulting in a 20% reduction of NO release at 50 μM and 100 μM concentrations. However, very little or no inhibition of NO release were seen at these concentrations for disaccharide **5** and trisaccharide **6** bearing an unnatural D-Fucα1-4GlcNAc linkage. Interestingly, glycosphingolipid **1** showed stronger activity than **2**, suggesting that the ceramide-based aglycon contributes to the inhibition of NO release more efficiently than a 2-(trimethylsilyl) ethyl-based aglycon. Moreover, commercial ceramide **7** showed inhibitory activity at higher concentration (100 μM). However, the activity of ceramide is strongly enhanced by glycosylation to the trisaccharide β-D-GalNAc*p*(1→4)[α-D-Fuc*p*(1→3)]-β-D-GlcNAc indicating that both the trisaccharide and ceramide-based aglycon portion of the glycosphingolipid contribute to the inhibition of cellular nitric oxide release.

## 3. Experimental

### 3.1. General

Optical rotations were measured with a Jasco P-1020 digital polarimeter. ^1^H- and ^13^C-NMR spectra were recorded with JMN A500 and ECP 600 FT NMR spectrometers with Me_4_Si as the internal standard for solutions in CDCl_3_ and CD_3_OD. MALDI-TOFMS was recorded on an Applied Biosystems Voyager DE RP mass spectrometer. High-resolution mass spectra were recorded on a JEOL JMS-700 under FAB conditions. TLC was performed on Silica Gel 60 F254 (E. Merck) with detection by quenching of UV fluorescence and by charring with 10% H_2_SO_4_. Column chromatography was carried out on Silica Gel 60 (E. Merck). The compounds 3,4,6-Tri-*O*-acetyl-2-deoxy-2-(2,2,2-trichloroethoxycarbonylamino)-β-D-galactopyranosyl-(1→4)- [2,3,4-tri-*O*-acetyl-α-D-fucopyranosyl-(1→3)]- 6-*O*-acetyl-2-deoxy-2-(2,2,2-trichloroethoxycarbonylamino)-D-glucopyranosyl trichloroacetimidate **(8)** [6], 2-(trimethylsilyl)ethyl 2,3,4,6-tetra-*O*-acetyl-β-D-galactopyranosyl-(1→4)-6-*O*-benzyl-2-deoxy-2-(2,2,2-trichloroethoxycarbonylamino)-β-D-glucopyranoside (**12**) [3], phenyl 2,3,4-tri-*O*-benzyl-1-thio-β-D-fucopyranoside (**13**) [6], 2-(Trimethylsilyl)ethyl 4,6-*O*-benzyl-idene-2-deoxy-2-(2,2,2-trichloroethoxycarbonylamino)-β-D-glucopyranoside (**16**) [6], 2-(trimethyl-silyl)ethyl 6-*O*-benzyl-3-*O*-chloroacetyl-2-deoxy-2-(2,2,2-trichloroethoxycarbonylamino)-β-D-gluco-pyranoside (**19**) [6] and 2-(trimethylsilyl)ethyl 6-*O*-benzyl-2-deoxy-2-phthalimide-β-D-gluco-pyranoside (**22**) [18] were prepared as reported. Benzylceramide **9** was prepared by the conventional four-steps procedure [15] from phytosphingosine, which was purchased from Degussa (The Netherlands). 

*3,4,6-Tri-O-acetyl-2-deoxy-2-(2,2,2-trichloroethoxycarbonylamino)-β-D-galactopyranosyl-(1→4)- [2,3,4-tri-O-acetyl-α-D-fucopyranosyl-(1→3)]-6-O-acetyl-2-deoxy-2-(2,2,2-trichloroethoxycarbonyl-amino)-β-D-glucopyranosyl-(1→1)-(2S,3S,4R)-3,4-di-O-benzyl-2-hexadecanamido-octadecane-3,4-di-ol* (**10**). Four Å molecular sieves (250 mg) were added to a solution of **8** (21 mg, 16.5 μmol) and (2*S*,3*S,*4*R*)-3-*O*-benzoyl-2-hexadecanamido-4-octa-decene-1,3-diol **9** (24 mg, 30.0 μmol) in dry CH_2_Cl_2_ (0.5 mL) and the mixture was stirred for 16 h at room temperature, then cooled to 0 °C. TMSOTf (3 μL, 0.01 mmol) was added, and the mixture was stirred for 1 h at 0 °C, then neutralized with Et_3_N. The solids were filtered off and washed with CHCl_3_. The combined filtrate and washings were successively washed with water, dried (MgSO_4_), and concentrated. The product was purified by silica gel column chromatography using 3:1 toluene-EtOAc as eluent to give **10** (10 mg, 33%). [α]_D_^23^ = +18.2°(*c* 1.0, CHCl_3_); ^1^H-NMR (500 MHz, CDCl_3_): δ 4.94 (d, 1H, *J*_1,2_=3.7Hz, H-1 of fuc), 4.50 (br. d, 1H, H-1 of GlcNAc), 4.34 (br. s, 1H, H-1, of GalNAc). MALDI-TOFMS: Calcd for C_86_H_129_Cl_6_N_3_O_27_Na [M+Na]^+^: *m/z* 1868.7 Found: 1869.4.

*2-Acetamido-3,4,6-tri-O-acetyl-2-deoxy-β-D-galactopyranosyl-(1→4)- [2,3,4-tri-O-acetyl-α-D-fucopyranosyl-(1→3)]-2-acetamido-6-O-acetyl-2-deoxy-β-D-glucopyranosyl-(1→1)-(2S,3S,4R)-hexa-decanamido-octadecane-3,4-di-ol* (**11**). To a solution of **10** (31mg, 16.8 μmol) in acetic anhydride (2 mL) and AcOH (2 mL) was added zinc powder (100 mg). The reaction mixture was stirred for 16 h at room temperature. After completion of the reaction, the solids were filtered off and the filtrate was concentrated with toluene. The solution of the product and Pd/C (10%, 100 mg) in 1:1 MeOH/THF (2.0 mL) was stirred for 16 h at room temperature under H_2_, then filtered and concentrated. The product was purified by silica gel column chromatography using 2:1 toluene acetone as eluent to give **11** as an amorphous powder (11 mg, 47%). [α]_D_^23^ = +5.3°(*c* 0.7, CHCl_3_); ^1^H-NMR (500 MHz, CDCl_3_): δ 5.15 (d, 1H, *J*_1,2_=3.7Hz, H-1 of fuc), 4.33 (br. d, 1H, H-1 of GlcNAc), 4.30 (br. s, 1H, H-1, of GalNAc). MALDI-TOFMS: Calcd for C_70_H_119_N_3_O_25_Na [M+Na]^+^: *m/z* 1424.8 Found: 1424.5.

*2-Acetamido-2-deoxy-β-D-galactopyranosyl-(1→4)-[α-D-fucopyranosyl-(1→3)]-2-acetamido-2-deoxy-β-D-glucopyranosyl-(1→1)-(2S,3S,4R)–hexadecanamido-octadecane-3,4-di-ol* (**1**). To a solution of **11** (11 mg, 7.8 μmol) in MeOH (2 mL) was added dioxane (2 mL) and NaOMe (25 mg) at 40 °C. The mixture was stirred for 2 h and then neutralized with Amberlite IR 120 [H^+^]. The mixture was filtered and concentrated. The product was purified by Sephadex LH-20 column chromatography in 1:1 CHCl_3_-MeOH to give **1** as white solid (10 mg, quant.). [α]_D_^25^ +17.0 (*c*=0.06, 1:1 CHCl_3_-MeOH).^1^H-NMR (500 MHz, 1:1 CDCl_3_-CD_3_OD): δ 5.13 (d, 1H, *J*=3.7Hz, H-1 of Fuc), 4.62 (d, 1H, *J* =8.3Hz, H-1 of GlcNAc), 4.32 (d, 1H, d, 1H, *J* =8.0Hz, H-1 of GalNAc). MALDI-TOFMS: Calcd for C_56_H_105_N_3_O_18_Na: *m/z* 1130.7 Found: 1130.4 [M+Na]^+^. HR-FABMS: Calcd for C_56_H_105_N_3_O_18_Na: *m/z* 1130.7291. Found *m/z* 1130.7257 [M+Na]^+^.

*2-(Trimethylsilyl)ethyl 2,3,4,6-tetra-O-acetyl-β-D-galactopyranosyl-(1→4)- [2,3,4-tri-O-benzyl-α-D-fucopyranosyl-(1→3)]-6-O-benzyl-2-deoxy-2-(2,2,2-trichloroethoxy- carbonylamino)-β-D-gluco-pyranoside* **(14)**. To a solution of **12** (99 mg, 0.11 mmol) and **13 **(89 mg, 0.17 mmol) in dry CH_2_Cl_2_ (1.5 mL) was added powdered MS 4Å (200 mg), and the mixture was stirred for 2 h at room temperature, then cooled to -60 °C. NIS (57 mg, 0.03 mmol) and TfOH (1.5 μL, 0.01 mmol) were added to the mixture, which was stirred for 3 h at -60 °C, then neutralized with Et_3_N. The solids were filtered off and washed with CHCl_3_. The combined filtrate and washings were successively washed with aq Na_2_S_2_O_3_ and water, dried (MgSO_4_), and concentrated. The product was purified by silica gel column chromatography using 3:1 hexane-EtOAc as eluent to give **14** (128 mg, 88%). [α]_D_^24^ +20.4 (*c* 0.7, CHCl_3_); ^1^H-NMR (500 MHz, CDCl_3_): δ 7.28–7.14 (m, 20H, 4Ph), 5.74 (d, 1H, NH), 5.26 (d, 1H, H-4 of Gal), 5.11 (t, 1H, H-2 of Gal), 4.92–4.84 (m, 2H, H-3 of Gal, benzylmethylene), 4.85 (d, 1H, *J*_1,2_=3.7 Hz, H-1 of Fuc), 4.75–4.54 (m, 7H, benzylmethylene × 5, CH_2_CCl_3_), 4.40 (d, 1H, *J*_1,2_=7.3 Hz, H-1 of GlcNAc), 4.38 (d, 1H, *J*_1,2_=7.9 Hz, H-1 of Gal), 4.27 (dd, 2H, benzylmethylene x 2), 4.06–4.02 (m, 2H, H-6a of GlcNAc, H-5 of Fuc), 3.97–3.75 (m, 9H, H-2, H-3, H-6b of GlcNAc, H-5, H-6 of Gal, H-2, H-3 of Fuc, CH_2_CH_2_Si(CH_3_)_3_), 3.66–3.62 (m, 3H, H-4, H-5 of GlcNAc, H-4 of Fuc), 3.38–3.32 (m, 1H, CH_2_CH_2_Si(CH_3_)_3_), 2.07–1.87 (m, 12H, CH_3_CO × 4), 1.09 (d, 3H, H-6 of Fuc), 0.88–0.73 (m, 2H, CH_2_CH_2_Si(CH_3_)_3_), –0.09 (s, 9H, Si(CH_3_)_3_); ^13^C-NMR (125 MHz, CDCl_3_): δ 170.2, 170.0, 154.2, 139.0, 138.9, 138.7, 138.3, 128.4, 128.23, 128.20, 127.8, 127.74, 127.65, 127.59, 127.5, 127.3, 100.2 (C-1 of GlcNAc), 100.1 (C-1 of Gal), 99.3 (C-1 of Fuc), 95.8, 79.5, 77.8, 76.4, 76.3, 75.6, 74.8, 74.6, 74.1, 73.5, 73.2, 72.9, 71.0, 70.4, 69.0, 67.4, 66.8, 66.7, 61.1, 53.6, 29.7, 20.8, 20.62, 20.57, 18.2, 16.5, –1.4 (Si(CH_3_)_3_); MALDI-TOFMS: Calcd for C_62_H_78_Cl_3_NO_20_SiNa: *m/z* 1312.4 Found: 1312.9 [M+Na]^+^.

*2-(Trimethylsilyl)ethyl 2,3,4,6-tetra-O-acetyl-β-D-galactopyranosyl-(1→4)- [2,3,4-tri-O-acetyl-α-D-fucopyranosyl-(1→3)]-6-O-acetyl-2-acetamido-2-deoxy-β-D-glucopyranoside* (**15**). To a solution of **14** (107 mg, 0.08 mmol) in acetic anhydride (6 mL) and AcOH (6 mL) was added zinc powder (150 mg). The reaction mixture was stirred for 12 h at 40 °C. After completion of the reaction, the solids were filtered off and the filtrate was concentrated with toluene. The solution of the product and Pd/C (10%, 100 mg) in MeOH (2.0 mL) was stirred for 16 h at room temperature under H_2_, then filtered and concentrated. The residue was acetylated with acetic anhydride (2 mL) in pyridine (3 mL) for 16 h at room temperature. The reaction mixture was poured into ice-water and extracted with CHCl_3_. The extract was washed sequentially with 5% HCl, aq NaHCO_3_ and water, dried (MgSO_4_), and concentrated. The product was purified by silica gel column chromatography using 5:1 toluene-acetone as eluent to give **15** (37 mg, 46%) as an amorphous powder. [α]_D_^24^ +11.8 (*c* 0.7, CHCl_3_); ^1^H-NMR (500 MHz, CDCl_3_): δ 6.47 (d, 1H, NH), 5.36 (d, 1H, H-4 of Gal), 5.23–5.21 (m, 2H, H-2 of Gal, H-4 of Fuc), 5.10–5.00 (m, 3H, H-3 of Gal, H-2, H-3 of Fuc), 5.08 (d, 1H, *J*_1,2_=3.7 Hz, H-1 of Fuc), 4.57 (dd, 1H, H-6a of Gal), 4.52 (d, 1H, *J*_1,2_=7.9 Hz, H-1 of Gal),4.42 (dd, 1H, H-5 of Fuc), 4.37 (d, 1H, *J*_1,2_=7.9 Hz, H-1 of GlcNAc), 4.33 (dd, 1H, H-6b of Gal), 4.24 (br d, 1H, H-2 of GlcNAc), 4.09 (d, 2H, H-6 of GlcNAc), 3.93–3.85 (m, 4H, H-3, H-4 of GlcNAc, H-5 of Gal, CH_2_CH_2_Si(CH_3_)_3_), 3.69(s, 1H, H-5 of GlcNAc), 2.18–1.81 (m, 27H, CH_3_CO × 9), 1.13 (d, 3H, H-6 of Fuc), 0.98–0.80 (m, 2H, CH_2_CH_2_Si(CH_3_)_3_), –0.02 (s, 9H, Si(CH_3_)_3_); ^13^C-NMR (125 MHz, CDCl_3_): δ 170.9, 170.7, 170.5, 170.3, 170.2, 170.0, 169.9, 169.5, 99.5 (C-1 of Gal), 99.4 (C-1 of GlcNAc), 97.3 (C-1 of Fuc), 73.4, 72.5, 71.8, 71.3, 71.2, 69.9, 68.8, 68.2, 67.9, 66.7, 66.3, 65.7, 64.9, 61.0, 48.5, 22.9, 21.0, 20.9, 20.7, 20.6, 20.52, 20.47, 17.9, 15.7, –1.5 (Si(CH_3_)_3_); MALDI-TOFMS: Calcd for C_41_H_63_NO_23_SiNa: *m/z* 988.3 Found: 988.4 [M+Na]^+^. 

*2-(Trimethylsilyl)ethyl* *β-D-galactopyranosyl-(1→4)-[α-D-fucopyranosyl-(1→3)]-2-acetamido-2-deoxy-β-D-glucopyranoside* (**3**). To a solution of **15** (36 mg, 0.04 mmol) in MeOH (5 mL) NaOMe (25 mg) was added at 40 °C. The mixture was stirred for 2 h and then neutralized with Amberlite IR 120 [H^+^]. The mixture was filtered and concentrated. The product was purified by Sephadex LH-20 column chromatography in 1 : 1 CHCl_3_-MeOH to give **5** as white solid (24 mg, quant.). [α]_D_^24^ +14.4 (*c* 0.3, CH_3_OH); ^1^H-NMR (500 MHz, CD_3_OD): δ 5.12 (d, 1H, *J*_1,2_=4.3 Hz, H-1 of Fuc), 4.42 (d, 1H, *J*_1,2_=7.9 Hz, H-1 of GlcNAc), 4.32 (d, 1H, *J*_1,2_=7.9 Hz, H-1 of Gal); ^13^C=NMR (125 MHz, CD_3_OD): δ 173.4, 104.1 (C-1 of GlcNAc), 102.2 (C-1 of Gal), 102.1 (C-1 of Fuc), 74.8, 73.6, 72.7, 71.5, 70.90, 70.86, 68.5, 67.9, 62.9, 61.1, 56.7, 30.7, 23.5, 18.8, 16.8, –1.3 (Si(CH_3_)_3_); MALDI-TOFMS: Calcd for C_25_H_47_NO_15_SiNa: *m/z* 652.3 Found: 652.6 [M+Na]^+^. HR-FABMS: Calcd for C_25_H_47_NO_15_SiNa: *m/z* 652.2613. Found *m/z* 652.2642 [M+Na]^+^.

*2-(Trimethylsilyl)ethyl 2,3,4-tri-O-benzyl-α-D-fucopyranosyl-(1→3)-4,6-O-benzylidene-2-deoxy-2-(2,2,2-trichloroethoxycarbonylamino)-β-D-glucopyranoside* (**17**). To a solution of **16** (329 mg, 0.61 mmol) and **13 **(479 mg, 0.91 mmol) in dry CH_2_Cl_2_ (1.5 mL) was added powdered 4Å MS (800 mg), and the mixture was stirred for 2 h at room temperature, then cooled to -60 °C. NIS (307 mg, 1.37 mmol) and TfOH (16 μL, 0.18 mmol) were added to the mixture, which was stirred for 3 h at -60 °C, then neutralized with Et_3_N. The solids were filtered off and washed with CHCl_3_. The combined filtrate and washings were successively washed with aq Na_2_S_2_O_3_ and water, dried (MgSO_4_), and concentrated. The product was purified by silica gel column chromatography using 7:1 hexane-EtOAc as eluent to give **17** (394 mg, 68%). [α]_D_^24^ +18.9 (*c* 2.4, CHCl_3_); ^1^H-NMR (500 MHz, CDCl_3_): δ 7.41–6.89 (m, 20H, 4Ph), 5.49 (br s, 1H, H-1 of Fuc), 5.31 (s, 1H, OCHPh), 5.14 (br s, 1H, NH), 4.86–4.75 (m, 3H, benzylmethylene × 2, CH_2_CCl_3_), 4.64 (d, 1H, *J*_1,2_=8.6 Hz, H-1 of GlcNAc), 4.60 (d, 1H, CH_2_CCl_3_), 4.48 (t, 2H, benzylmethylene × 2), 4.36–4.29 (m, 2H, benzylmethylene × 2), 4.25–4.18 (m, 2H, H-3, H-6a of GlcNAc), 3.96–3.85 (m, 3H, CH_2_CH_2_Si(CH_3_)_3_, H-2, H-5 of Fuc), 3.81–3.67 (m, 3H, H-4, H-6b of GlcNAc, H-3 of Fuc), 3.52–3.40 (m, 4H, CHCH_2_Si(CH_3_)_3_, H-2, H-5 of GlcNAc, H-4 of Fuc), 1.01 (d, 3H, H-6 of Fuc), 0.94–0.79 (m, 2H, CH_2_CH_2_Si(CH_3_)_3_), –0.07 (s, 9H, Si(CH_3_)_3_); ^13^C-NMR (125 MHz, CDCl_3_): δ 153.7, 138.8, 138.4, 138.2, 136.9, 129.3, 128.5, 128.34, 128.27, 128.1, 128.0, 127.5, 127.3, 127.1, 126.2, 101.6, 100.8 (C-1 of GlcNAc), 97.0 (C-1 of Fuc), 95.4, 82.5, 78.6, 75.1, 74.8, 74.4, 73.6, 73.3, 71.4, 68.7, 67.6, 67.0, 65.8, 57.0, 29.6, 18.2, 16.7, –1.5 (Si(CH_3_)_3_); MALDI-TOFMS: Calcd for C_48_H_58_Cl_3_NO_11_SiNa [M+Na]^+^: *m/z* 980.3. Found: 980.1.

*2-(Trimethylsilyl)ethyl 2,3,4-tri-O-acetyl-α-D-fucopyranosyl-(1→3)-2-acetamido-4,6-di-O-acetyl-2-deoxy-β-D-glucopyranoside* (**18**). To a solution of **17** (113 mg, 0.12 mmol) in acetic anhydride (7 mL) and AcOH (7 mL) was added zinc powder (150 mg). The reaction mixture was stirred for 12 h at 40 °C. After completion of the reaction, the solids were filtrered off and the filtrate was concentrated with toluene. The solution of the product and Pd/C (10%, 150 mg) in 3:1 MeOH-AcOH (2.0 mL) was stirred for 12 h at room temperature under H_2_, then filtered and concentrated. The residue was acetylated with acetic anhydride (6 mL) in pyridine (10 mL) for 12 h at room temperature. The reaction mixture was poured into ice-water and extracted with CHCl_3_. The extract was washed sequentially with 5% HCl, aq NaHCO_3_ and water, dried (MgSO_4_), and concentrated. The product was purified by silica gel column chromatography using 5:1 toluene-acetone as eluent to give **18** (35 mg, 44%)as an amorphous powder. [α]_D_^24^ +74.9 (*c* 0.3, CHCl_3_); ^1^H-NMR (500 MHz, CDCl_3_): δ 5.85 (d, 1H, NH), 5.25 (d, 1H, *J*_1,2_=3.7 Hz, H-1 of Fuc), 5.26–5.23 (m, 2H, H-3, H-4 of Fuc), 5.08 (dd, 1H, H-2 of Fuc), 5.06 (d, 1H, *J*_1,2_=7.9 Hz H-1 of GlcNAc), 4.97 (dd, 1H, H-4 of GlcNAc), 4.67 (t, 1H, H-3 of GlcNAc), 4.25 (dd, 1H, H-5 of Fuc), 4.14 (dd, 1H, H-6a of GlcNAc), 3.99 (dd, 1H, H-6b of GlcNAc), 3.92–3.87 (m, 1H, CH_2_CH_2_Si(CH_3_)_3_), 3.61–3.51 (m, 2H, H-5 of GlcNAc, CH_2_CH_2_Si(CH_3_)_3_), 3.09–3.04 (m, 1H, H-2 of GlcNAc), 1.08 (d, 3H, H-6 of Fuc), 0.96–0.83 (m, 2H, CH_2_CH_2_Si(CH_3_)_3_), -0.02 (s, 9H, Si(CH_3_)_3_); ^13^C-NMR (125 MHz, CDCl_3_): δ 170.7, 170.5, 98.1 (C-1 of GlcNAc), 95.8 (C-1 of Fuc), 73.7, 72.3, 71.5, 71.0, 67.6, 67.4, 67.2, 64.9, 62.5, 58.1, 23.8, 20.9, 20.8, 20.6, 18.1, 16.1 -1.4 (Si(CH_3_)_3_); MALDI-TOFMS: Calcd for C_29_H_47_NO_15_SiNa [M+Na]^+^: *m/z* 700.3 Found: 700.5.

*2-(Trimethylsilyl)ethyl* *α-D-fucopyranosyl-(1→3)-2-acetamido-2-deoxy-β-D-glucopyranoside* (**4**). Compound **4** was prepared from **18** (24 mg, 0.035 mmol) by the same method described for preparation of **3**. The product was purified by Sephadex LH-20 column chromatography in 1:1 CHCl_3_-MeOH to give **4** as white solid (14 mg, 86%). [α]_D_^24^ +52.9 (*c* 0.1, CH_3_OH); ^1^H-NMR (500 MHz, CD_3_OD): δ 4.93 (d, 1H, *J*_1,2_=3.1 Hz, H-1 of Fuc), 4.30 (d, 1H, *J*_1,2_=8.5 Hz H-1 of GlcNAc); ^13^C-NMR (125 MHz, CD_3_OD): δ 173.3, 103.6 (C-1 of GlcNAc), 102.2 (C-1 of Fuc), 86.3, 79.5, 77.4, 73.7, 72.5, 71.6, 70.8, 68.5, 67.9, 62.6, 60.2, 55.9, 30.7, 23.4, 18.8, 16.9, -1.26 (Si(CH_3_)_3_); MALDI-TOFMS: Calcd for C_19_H_37_NO_10_SiNa: *m/z* 490.2 Found: 490.6 [M+Na]^+^. HR-FABMS: Calcd for C_19_H_37_NO_10_SiNa: *m/z* 490.2084. Found *m/z* 490.2072 [M+Na]^+^.

*2-(Trimethylsilyl)ethyl 2,3,4-tri-O-benzyl-α-D-fucopyranosyl-(1→4)-6-O-benzyl-3-O-chloroacetyl-2-deoxy-2-(2,2,2-trichloroethoxycarbonylamino)-β-D-glucopyranoside*(**20**). Compound **20** was prepared from **19** (226 mg, 0.36 mmol) and **13** (383 mg, 0.73 mmol) by the same method described for preparation of **17**. The product was purified by silica gel column chromatography using10:1 hexane-EtOAc as eluent to give **20** as syrup (296 mg, 78%). [α]_D_^24^ +11.4 (*c* 4.0, CHCl_3_); ^1^H-NMR (500 MHz, CDCl_3_): δ 7.37–7.10 (m, 20H, 4 Ph), 5.49 (d, 1H, NH), 5.22 (t, 1H, H-3 of GlcNAc), 4.89 (d, 1H, benzylmethylene), 4.87 (d, 1H, *J*_1,2_=3.7 Hz, H-1 of Fuc), 4.76–4.53 (m, 9H, H-4 of Fuc, benzylmethylene × 6, CH_2_CCl_3_), 4.50 (d, 1H, *J*_1,2_=7.3 Hz, H-1 of GlcNAc), 4.45 (d, 1H, benzylmethylene), 3.92–3.79 (m, 5H, H-4, 6a of GlcNAc, H-2, 5 of Fuc, CH_2_CH_2_Si(CH_3_)_3_), 3.75–3.62 (m, 5H, H-2, H-6b of GlcNAc, H-3 of Fuc, ClCH_2_CO), 3.51–3.46 (m, 2H, CH_2_CH_2_Si(CH_3_)_3_, H-5 of GlcNAc), 0.97 (d, 3H, H-6 of Fuc), 0.98_2_0.83 (m, 2H, CH_2_CH_2_Si(CH_3_)_3_), –0.09 (s, 9H, Si(CH_3_)_3_); ^13^C-NMR (125 MHz, CDCl_3_): δ 167.1, 154.1, 138.5, 138.2, 138.1, 128.6, 128.43, 128.36, 128.3, 128.1, 127.62, 127.55, 127.47, 127.37, 100.1(C-1 of Fuc), 98.8(C-1 of GlcNAc), 95.5, 78.9, 77.7, 75.6, 74.9, 74.8, 74.6, 74.2, 74.0, 73.3, 73.2, 68.7, 67.6, 67.1, 55.5, 40.8, 18.1, 16.6, –1.32 (Si(CH_3_)_3_); MALDI-TOFMS: Calcd for C_50_H_61_Cl_4_NO_12_SiNa [M+Na]^+^: *m/z* 1058.3 Found: 1059.2.

*2-(Trimethylsilyl)ethyl 2,3,4-tri-O-acetyl-α-D-fucopyranosyl-(1→4)-2-acetamido-3,6-di-O-acetyl-2-deoxy-β-D-glucopyranoside* (**21**). To a solution of **20** (296 mg, 0.29 mmol) in EtOH (2.5 mL) was added pyridine (1.5 mL) and thiourea (173 mg, 2.32 mmol). The reaction mixture was stirred for 6 h at 80 °C. The mixture was diluted with CHCl_3_, washed with aq 5%HCl, aq NaHCO_3_ and brine, dried (MgSO_4_) and concentrated. The solution of the residue in AcOH (2 mL) was added zinc powder (350 mg). The reaction mixture was stirred for 12 h at 60 °C. After completion of the reaction, the solids were filtered off and the filtrate was concentrated with toluene. The residue was acetylated with acetic anhydride (4 mL) in pyridine (7 mL). The reaction mixture was poured into ice-water and extracted with CHCl_3_. The extract was washed sequentially with 5% HCl, aq. NaHCO_3_ and water, dried (MgSO_4_), and concentrated. The solution of the product in MeOH (1.5 mL) and THF (0.5 mL) was hydrogenolysed under hydrogen in the presence of 10% Pd/C (150 mg) for 16 h at room temperature, then filtered and concentrated. The residue was acetylated with acetic anhydride (3 mL) in pyridine (5 mL). The reaction mixture was poured into ice-water and extracted with CHCl_3_. The extract was washed sequentially with 5% HCl, aq NaHCO_3_ and water, dried (MgSO_4_), and concentrated. The product was purified by silica gel column chromatography using 5:1 toluene-acetone as eluent to give **21** as an amorphous powder (86 mg, 45%). [α]_D_^24^ +49.6 (*c* 0.5, CHCl_3_); ^1^H-NMR (500 MHz, CDCl_3_): δ 5.81 (d, 1H, NH), 5.34 (d, 1H, *J*_1,2_=3.7 Hz, H-1 of Fuc), 5.22–5.05 (m, 4H, H-3 of GlCNAc, H-2,H-3, H-4 of Fuc), 4.58 (d, 1H, *J*_1,2_=7.9 Hz H-1 of GlcNAc), 4.46 (dd, 1H, H-6a of GlcNAc), 4.08–4.01 (m, 2H, H-6b of GlcNAc, H-5 of Fuc), 3.95 (t, 1H, H-4 of GlcNAc), 3.88–3.83 (m, 1H, CH_2_CH_2_Si(CH_3_)_3_), 3.78 (dd, 1H, H-2 of GlcNAc), 3.61–3.57 (m, 1H, H-5 of GlcNAc), 3.53–3.48 (m, 1H, CH_2_CH_2_Si(CH_3_)_3_), 2.11–1.82 (m, 18H, CH_3_CO × 6), 1.04 (d, 3H, H-6 of Fuc), 0.91–0.78 (m, 2H, CH_2_CH_2_Si(CH_3_)_3_), –0.07 (s, 9H, Si(CH_3_)_3_); ^13^C-NMR (125 MHz, CDCl_3_): δ 171.0, 170.7, 170.5, 170.3, 170.1, 170.0, 99.8 (C-1 of GlcNAc), 96.0 (C-1 of Fuc), 75.5, 72.1, 71.8, 70.9, 67.3, 67.2, 66.9, 65.5, 62.7, 54.6, 29.6, 23.1, 20.9, 20.8, 20.7, 20.6, 20.5, 17.8, 15.8, –1.5 (Si(CH_3_)_3_); MALDI-TOFMS: Calcd for C_29_H_47_NO_15_SiNa [M+Na]^+^: *m/z* 700.3 Found: 700.9.

*2-(Trimethylsilyl)ethyl* *α-D-fucopyranosyl-(1→4)-2-acetamido-2-deoxy-β-D-glucopyranoside* (**5**). Compound **5** was prepared from **21** (86 mg, 0.13 mmol) by the same method described for preparation of **3**. The product was purified by Sephadex LH-20 column chromatography in 1:1 CHCl_3_-MeOH to give **5** as white solid (61 mg, quant.). [α]_D_^24^ +33.9 (*c* 0.3, CH_3_OH); ^1^H-NMR (500 MHz, CD_3_OD): δ 4.98 (d, 1H, *J*_1,2_=3.7 Hz, H-1 of Fuc), 4.32 (d, 1H, *J*_1,2_=7.9 Hz H-1 of GlcNAc); ^13^C-NMR (125 MHz, CD_3_OD): δ 173.5, 103.5 (C-1 of Fuc), 102.0 (C-1 of GlcNAc), 82.2, 76.9, 75.9, 73.5, 71.7, 70.6, 68.6, 67.9, 62.5, 56.8, 30.7, 23.0, 18.8, 16.7, –1.3 (Si(CH_3_)_3_); MALDI-TOFMS: Calcd for C_19_H_37_NO_10_SiNa: *m/z* 490.2 Found: 491.0 [M+Na]^+^. HR-FABMS: Calcd for C_19_H_37_NO_10_SiNa: *m/z* 490.2084. Found *m/z* 490.2062 [M+Na]^+^.

*2-(Trimethylsilyl)ethyl 2,3,4-tri-O-benzyl-α-D-fucopyranosyl-(1→3)- [2,3,4-tri-O-benzyl-α-D-fuco-pyranosyl-(1→4)]-6-O-benzyl-2-deoxy-2-phthalimido-β-D-glucopyranoside* (**23**). Compound **23** was prepared from **22** (89 mg, 0.18 mmol) and **13** (757 mg, 1.44 mmol) by the same method described for preparation of **14**. The product was purified by silica gel column chromatography using 10:1 hexane- EtOAc as eluent to give **23** as syrup (99 mg, 42%). [α]_D_^24^ +48.3 (*c* 1.2, CHCl_3_); ^1^H-NMR (600 MHz, CDCl_3_): δ 7.90–7.26 (m, 39H, NPhth, 8 Ph), 6.20 (d, 1H, *J*_1,2_=3.6 Hz, H-1 of Fuc b), 5.19 (d, 2H, *J*_1,2_=8.5 Hz H-1 of GlcNAc, *J*_1,2_=4.4 Hz H-1 of Fuc a), 5.07 (d, 1H, benzylmethylene), 5.01 (dd, 1H, H-3 of GlcNAc), 4.93–4.88(m, 3H, benzylmethylene × 3), 4.79–4.66 (m, 8H, benzylmethylene × 8), 4.62–4.58 (m, 2H, benzylmethylene × 2), 4.41 (dd, 1H, H-2 of GlcNAc), 4.18–4.13 (m, 2H, H-4 of GlcNAc, H-2 of Fuc b), 4.10 (dd, 1H,H-3 of Fuc a), 4.07–3.99 (m, 2H, CH_2_CH_2_Si(CH_3_)_3_, H-5 of Fuc b), 3.97–3.84 (m, 6H, H-5, H-6 of GlcNAc, H-2, H-5 of Fuc a, H-3 of Fuc b), 3.63–3.57 (m, 2H, H-4 of Fuc b, CH_2_CH_2_Si(CH_3_)_3_), 3.47(br.d, 1H, H-4 of Fuc a), 1.19 (d, 3H, H-6 of Fuc b) 0.95 (d, 3H, H-6 of Fuc a,), 0.93–0.81 (m, 2H, CH_2_CH_2_Si(CH_3_)_3_), –0.01 (s, 9H, Si(CH_3_)_3_); ^13^C-NMR (150 MHz, CDCl_3_): δ 139.1, 138.7, 138.52, 138.50, 138.3, 133.8, 128.3, 128.23, 128.18, 128.12, 128.05, 128.03, 127.93, 127.87, 127.7, 127.6, 127.5, 127.42, 127.37, 127.35, 127.28, 127.23, 127.1, 123.1, 169.3, 9, 97.6 (C-1 of GlcNAc), 97.5 (C-1 of Fuc a), 95.0 (C-1 of Fuc b), 78.8, 78.7, 78.4, 78.1, 78.0, 76.4, 76.3, 74.9, 74.6, 73.5, 73.3, 73.1, 73.0, 72.9, 72.8, 69.7, 67.7, 67.0, 66.7, 56.3, 29.7, 17.8, 16.6, 15.8, –1.5 (Si(CH_3_)_3_); MALDI-TOFMS: Calcd for C_80_H_89_NO_15_SiNa [M+Na]^+^: *m/z* 1354.6 Found: 1354.8.

*2-(Trimethylsilyl)ethyl 2,3,4-tri-O-acetyl-α-D-fucopyranosyl-(1→3)- [2,3,4-tri-O-acetyl-α-D-fuco-pyranosyl-(1→4)]- 2-acetamido-6-O-acetyl-2-deoxy-β-D-glucopyranoside* (**24**). To a solution of **23** (60 mg, 0.05 mmol in EtOH (10 mL)) was added hydrazine monohydrate (3.3 mL, 0.07 mmol). The reaction mixture was refluxed for 3 h, then concentrated. The residue was acetylated with Ac_2_O (3 mL) in pyridine (5 mL). The mixture was poured into ice-water and extracted with CHCl_3_. The extract was washed sequentially with 5% HCl, aq NaHCO_3_ and water, dried (MgSO_4_), and concentrated. The solution of the product in MeOH (1 mL) and THF (1 mL) was hydrogenolysed under hydrogen in the presence of 10% Pd/C (100 mg) for 15 h at room temperature, then filtered and concentrated. The residue was acetylated with acetic anhydride (5 mL) in pyridine (7 mL). The reaction mixture was poured into ice-water and extracted with CHCl_3_. The extract was washed sequentially with 5% HCl, aq NaHCO_3_ and water, dried (MgSO_4_), and concentrated. The product was purified by silica gel column chromatography using 9:1 toluene-acetone as eluent to give **24** as syrup (19 mg, 47%). [α]_D_^24^ +75.2 (*c* 0.5 CHCl_3_); ^1^H-NMR (500 MHz, CDCl_3_): δ 6.43 (d, 1H, NH), 5.28 (dd, 1H, H-3 of Fuc b), 5.23–5.18 (m, 3H, H-3, H-4 of Fuc a, H-4 of Fuc b), 5.13 (d, 1H, *J*_1,2_=3.7 Hz, H-1 of Fuc b), 5.08–5.03 (m, 2H, H-3 of GlcNAc, H-2 of Fuc b), 5.04 (d, 1H, *J*_1,2_=9.8 Hz H-1 of GlcNAc), 4.66 (d, 1H, *J*_1,2_=3.7 Hz H-1 of Fuc a), 4.52 (dd, 1H, H-6a of GlcNAc), 4.43–4.36 (m, 2H, H-6b of GlcNAc, H-5 of Fuc b), 4.13–4.08 (m, 2H, H-2 of GlcNAc, H-5 of Fuc a), 3.96 (t, 1H, H-5 of GlcNAc), 3.91–3.85 (m, 1H, CH_2_CH_2_Si(CH_3_)_3_), 3.60–3.55 (m, 2H, H-4 of GlcNAc, H-2 of Fuc a), 3.46–3.40 (m, 1H, CH_2_CH_2_Si(CH_3_)_3_), 2.13–1.87 (m, 21H, CH_3_CO) 1.10 (d, 3H, H-6 of Fuc b) 1.07 (d, 3H, H-6 of Fuc a), 0.95–0.80 (m, 2H, CH_2_CH_2_Si(CH_3_)_3_), –0.03 (s, 9H, Si(CH_3_)_3_); ^13^C-NMR (125 MHz, CDCl_3_): δ 170.5, 170.43, 170.36, 170.31, 169.9, 169.5, 169.3, 98.5 (C-1 of Fuc a), 98.2 (C-1 of Fuc b), 97.6 (C-1 of GlcNAc), 76.9, 76.8, 76.7, 74.1, 74.0, 73.7, 73.4, 71.2, 70.9, 68.3, 68.00, 67.97, 67.3, 66.5, 65.8, 65.7, 64.5, 49.3, 29.7, 23.4, 21.4, 20.9, 20.7, 20.6, 18.0, 16.0, 15.8, 15.7, 14.1, –1.5 (Si(CH_3_)_3_); MALDI-TOFMS: Calcd for C_39_H_61_NO_21_SiNa [M+Na]^+^: *m/z* 930.3 Found: 930.5.

*2-(Trimethylsilyl)ethyl* *α-D-fucopyranosyl-(1→3)-[ α-D-fucopyranosyl-(1→4)]- 2-acetamido-2-deoxy-β-D-glucopyranoside* (**6**). Compound **6** was prepared from **24** (16 mg, 0.03 mmol) by the same method described for preparation of **3**. The product was purified by Sephadex LH-20 column chromatography in 1:1 CHCl_3_-MeOH to give **6** as white solid (10 mg, 93%). [α]_D_^24^ +72.2 (*c* 0.2 CH_3_OH); ^1^H-NMR (600 MHz, CD_3_OD): δ 4.97 (d, 1H, *J*_1,2_=3.9 Hz H-1 of Fuc b), 4.66 (d, 1H, *J*_1,2_=2.8 Hz H-1 of Fuc a), 4.38 (d, 1H, *J*_1,2_=6.1 Hz H-1 of GlcNAc), –0.09 (s, 9H, Si(CH_3_)_3_); ^13^C-NMR (150 MHz, CD_3_OD): δ 172.8, 101.8 (C-1 of GlcNAc), 101.6 (C-1 of Fuc a), 100.1 (C-1 of Fuc b), 79.3, 78.3, 74.8, 73.7, 73.5, 71.4, 70.1, 69.8, 68.5, 68.3, 67.6, 63.1, 54.7, 27.0, 23.1, 18.9, 16.7, –1.3 (Si(CH_3_)_3_); MALDI-TOFMS: Calcd for C_25_H_47_NO_14_SiNa: *m/z* 636.3 Found: 636.7 [M+Na]^+^. HR-FABMS: Calcd for C_25_H_47_NO_14_SiNa: *m/z* 636.2664. Found *m/z* 636.2681 [M+Na]^+^.

### 3.2. Nitric Oxide Inhibitory Assay

J774.1 cells were grown in Dulbecco’s Modified Eagle’s Medium (DMEM, GIBCO) and cultured at 37°C in humidified 5% CO_2_/95% air. The cells were suspended in medium, plated on 96-well culture plates (Falcon) at a density of 5.0 × 10^5^ cells/mL/well, volume of 200 μL/well and allowed to adhere for 24 h. Then, the medium was replaced with fresh medium, containing LPS (1 μg/mL) from *E. coli* (Sigma) and test compounds dissolved in DMSO at various concentrations (13, 25, 50, 100 μM) were incubated for 24 h. NO production was determined by measuring the accumulation of nitrite (a stable metabolite of NO) in the culture supernatant using Griess reagent [20]. Briefly, 50 μL of the supernatant from incubates were mixed with equal volume of Griess reagent (1% sulfanilamide and 0.1% *N*-1-naphthylenediamine dihydrochloride in 5% H_3_PO_4_) and were allowed to stand for 10 minutes at room temperature. Absorbance at 550 nm was measured using a MTP-810 Microplate Reader (Corona Co.). The blank correction was carried out by subtracting the absorbance due to medium from the absorbance reading of each well. The reaction percentage was calculated as follows: % of control = [As/Ac] × 100, where As and Ac are absorbance of a run treated with LPS and a sample, and that treated with LPS alone, respectively. In this assay, N^G^-monomethyl-L-arginine (L-NMMA, IC_50_ 32.0 μM), a non-selective nitric oxide synthase (NOS) inhibitor, was used as a positive control [21].

## 4. Conclusions

We have succeeded for the first time in carrying out the total syntheses of D-fucose-containing glycosphingolipids found in invertebrate species. Both the presence of a D-Fucα1-3GlcNAc-linkage and the ceramide aglycon portion resulted in a significant enhancement of their ability to inhibit NO production by LPS-induced macrophage-like J774.1 cells. The prepared glycolipids are easily-accessible target compounds in the field of carbohydrate chemistry and may serve as chemical probes to explore glycosphingolipid-mediated anti-inflammatory processes in biology and medicine. 
